# Promising antibacterial efficacy of arenicin peptides against the emerging opportunistic pathogen *Mycobacterium abscessus*

**DOI:** 10.1186/s12929-024-01007-8

**Published:** 2024-01-29

**Authors:** Magali Casanova, Marc Maresca, Isabelle Poncin, Vanessa Point, Hamza Olleik, Céline Boidin-Wichlacz, Aurélie Tasiemski, Kamel Mabrouk, Jean-François Cavalier, Stéphane Canaan

**Affiliations:** 1 CNRS, Aix-Marseille Univ, LISM UMR7255, IMM FR3479, Marseille, France; 2 Aix Marseille Univ, CNRS, Centrale Marseille, iSm2 (UMR7313), Marseille, France; 3 Univ. Lille, CNRS, Inserm, CHU Lille, Institut Pasteur de Lille, U1019 - UMR9017 - CIIL - Center for Infection and Immunity of Lille, 59000 Lille, France; 4 Aix-Marseille Univ, CNRS, UMR7273, ICR, 13013 Marseille, France

**Keywords:** Mycobacterial infection, Cystic fibrosis, Hemolytic activity, Pore forming activity, Electron microscopy, Antibiotic resistance, Bactericidal activity, Antimicrobial peptide

## Abstract

**Background:**

*Mycobacterium abscessus*, a fast-growing non-tuberculous mycobacterium, is an emerging opportunistic pathogen responsible for chronic bronchopulmonary infections in people with respiratory diseases such as cystic fibrosis (CF). Due to its intrinsic polyresistance to a wide range of antibiotics, most treatments for *M. abscessus* pulmonary infections are poorly effective. In this context, antimicrobial peptides (AMPs) active against bacterial strains and less prompt to cause resistance, represent a good alternative to conventional antibiotics. Herein, we evaluated the effect of three arenicin isoforms, possessing two or four Cysteines involved in one (**Ar-1**, **Ar-2**) or two disulfide bonds (**Ar-3**), on the in vitro growth of *M. abscessus*.

**Methods:**

The respective disulfide-free AMPs, were built by replacing the Cysteines with alpha-amino-*n*-butyric acid (Abu) residue. We evaluated the efficiency of the eight arenicin derivatives through their antimicrobial activity against *M. abscessus* strains, their cytotoxicity towards human cell lines, and their hemolytic activity on human erythrocytes. The mechanism of action of the **Ar-1** peptide was further investigated through membrane permeabilization assay, electron microscopy, lipid insertion assay via surface pressure measurement, and the induction of resistance assay.

**Results:**

Our results demonstrated that **Ar-1** was the safest peptide with no toxicity towards human cells and no hemolytic activity, and the most active against *M. abscessus* growth. **Ar-1** acts by insertion into mycobacterial lipids, resulting in a rapid membranolytic effect that kills *M. abscessus* without induction of resistance.

**Conclusion:**

Overall, the present study emphasized **Ar-1** as a potential new alternative to conventional antibiotics in the treatment of CF-associated bacterial infection related to *M. abscessus*.

## Background

*Mycobacterium abscessus* is a non-tuberculous mycobacterium whose incidence is increasing worldwide, and which in some industrialized countries even exceeds that of *M. tuberculosis*, the etiological agent of tuberculosis [1]. *Mycobacterium abscessus* is responsible for severe lung infections and is associated with acute respiratory failure, notably in immunocompromised patients and/or patients with respiratory disorders such as cystic fibrosis (CF) [2, 3]. It is also responsible for cutaneous, bone and disseminated infections, and, in rare cases, for causing damage to the central nervous system [1]. Furthermore, the presence of *M. abscessus* may evolve into severe complications after lung transplantation, limiting the therapeutic arsenal offered to CF patients [4]. *Mycobacterium abscessus* exists as two phenotypically different morphotypes: a smooth (S) variant with a uniform, round and glossy appearance and that possesses glycopeptidolipids (GPL) at its surface; and an irregular and dry variant, called rough (R) variant, with no GPL at its surface thus resulting in cord formation [5, 6]. The S variant is associated with biofilm-like structures formation and should be responsible for the primo infection, whereas the R variant is usually found in severe and persistent forms of the clinical disease and represent the most virulent form of the bacteria [2, 7]. In addition, the naturally polyresistance of *M. abscessus*, not only to conventional anti-tuberculous drugs but also to most classes of antibiotics, including macrolides, aminoglycosides, rifamycins, tetracyclines, and β-lactams [1], leads to a treatment failure rate of nearly 60% [8]. Whether intrinsic, adaptive or acquired, *M. abscessus* resistance to many antibiotics involves several mechanisms: low permeability of the cell wall, induction of antibiotic efflux pumps, mutations inactivating antibiotic-activating enzymes or activation of enzymes that neutralize antibiotics or modify their targets [9, 10]. Consequently, in order to avoid bacterial resistance, the treatment of *M. abscessus* infections implements a multi-therapy possibly combined with surgery, resulting in an expensive and heavy treatment for the patient [11]. New drugs as well as new strategies are thus urgently needed to fight infections due to *M. abscessus*, ideally with a different mechanism of action than conventional antibiotics in order to minimize and prevent resistance.

In this context, some antimicrobial peptides (AMPs) have demonstrated particularly interesting antibacterial activities against *M. abscessus* [12–19]. AMPs are small-sized molecules (≤ 100 amino acids), with a cationic and a hydrophobic part [20]. They play function in the symbiostasis and in the microbial clearance of a large variety of organisms, from unicellular (bacteria, archaea) as anti-competitors, to pluricellular (plants, fungi, invertebrates and vertebrates) as key components of the innate immune response [20, 21]. Often constituting the first line of defense, they possess a killing activity against a wide range of microorganisms from Gram-negative and Gram-positive bacteria, to fungi, enveloped viruses as well as parasites [22–24]. More precisely, through their cationic part, AMPs interact with the anionic components at the surface of microorganisms, facilitating the insertion of the hydrophobic domain into the phospholipid bilayer thus inducing membrane destabilization and permeabilization resulting in bacterial lysis [20, 21]. AMPs can also target key intracellular processes that lead to pathogenic microorganism death without necessarily disrupting the membrane. Such processes include the inhibition of cell wall components expression, DNA or protein synthesis, protein folding, and metabolic turnover [25]. Besides having a direct antimicrobial activity, several AMPs also possess the ability to modulate the host innate immune response and thereby indirectly to promote intracellular pathogen clearance [26]. Given such diverse modes of action, few pathogens have developed resistance to AMPs which are also found active against pathogens already resistant to conventional antibiotics [27–30].

**Fig. 1.**
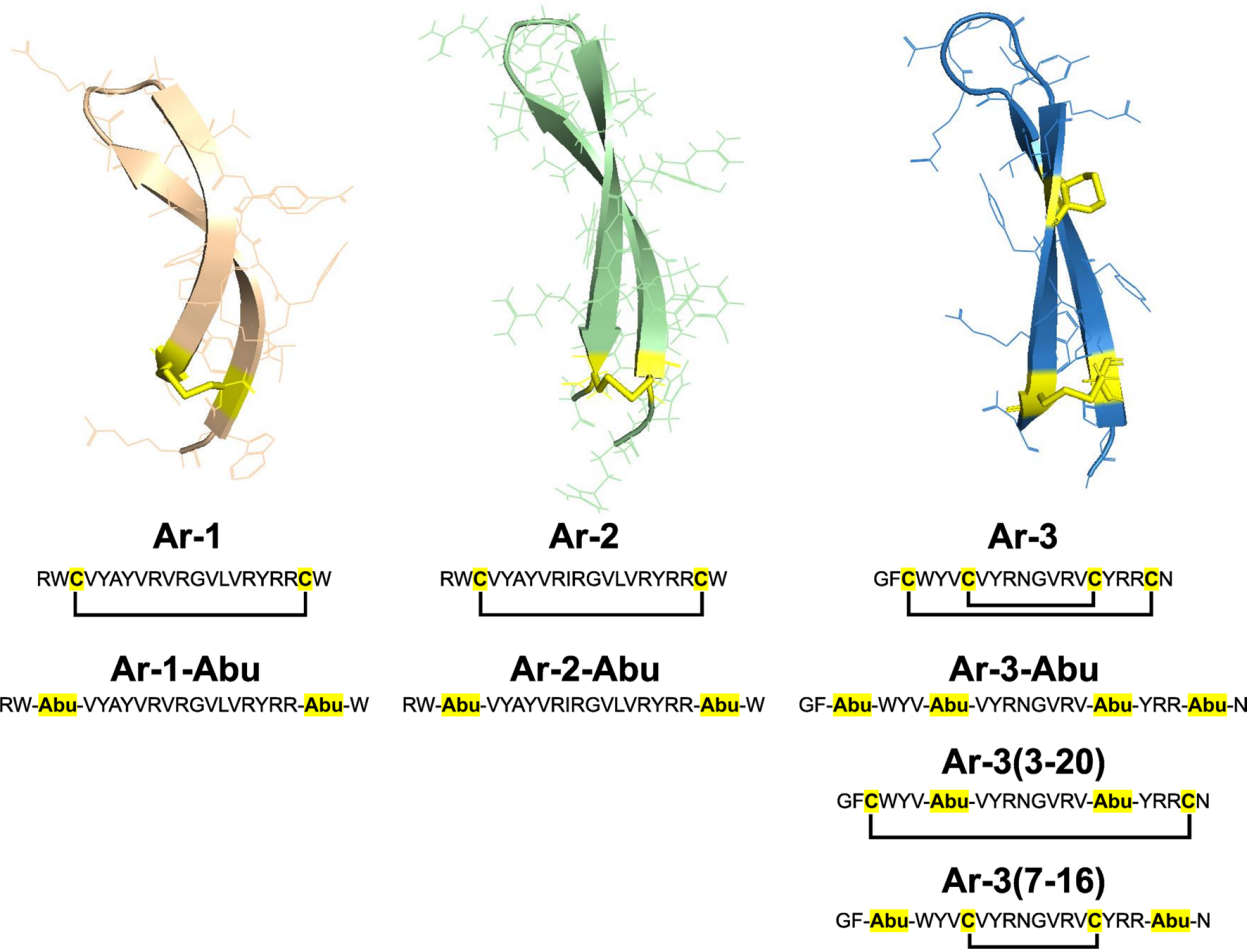
3-dimensional structures of arenicin peptides. Secondary structure of **Ar-1** (left, PDB id: 2JSB [32]), **Ar-2** (center, PDB id: 2JNI [36]) and **Ar-3** (right, PDB id: 5VOY [35]) represented as cartoons displaying anti-parallel β-sheets stabilized by disulfide bond(s). Below are reported their respective amino acid sequences showing cysteines highlighted in yellow and disulfide bond connectivity; as well as the disulfide-free derivatives **Ar-1-Abu**, **Ar-2-Abu**, **Ar-3-Abu** in which all cysteines were replaced by alpha-amino-*n*-butyric acid (Abu) residues, and **Ar-3(3–20)** and **Ar-3(7–16)** in which cysteines 1–16 and cysteines 3–20 were replaced by Abu residues, respectively

Arenicins (**Ar**) are AMPs of 21 residues that have been biochemically isolated and identified from circulating blood cells of a marine annelid worm called *Arenicola marina*, inhabiting the intertidal zone of the European temperate shore [31]. Three isoforms (**Ar-1**, **Ar-2** and **Ar-3**—Fig. 1) have been described, consisting in two antiparallel β-strands containing charged arginine residues as well as hydrophobic residues, explaining the amphipathic properties of these peptides [32–36].

Arenicins exhibit antifungal and antibacterial activities against Gram-negative and Gram-positive bacteria including drug-resistant clinical isolates [33–35]. They induce cell death in fungi as well as in bacteria via membrane permeabilization and disruption by formation of toroidal pores [33]. It has been recently demonstrated that **Ar-1** is also able to induce an apoptosis-like response in *Escherichia coli*-treated cells through accumulation of reactive oxygen species, imbalance of intracellular calcium concentrations, disruption of membrane potential, caspase-like activation, and DNA damage [37]. Another important issue for the therapeutic potential of AMPs is related to their anti-inflammatory activity. Their potential role in inflammation may indeed result from the regulation of various processes such as cytotoxicity towards eukaryotic cells, complement activation, steroid synthesis, mast cell activation, monocyte chemo-attraction, and enhancement of cytokine expression [38]. In the case of **Ar-1**, this peptide has been found to modulate complement activity in vitro depending on its concentration: stimulation at low concentrations (3.5–14.5 µM) and acting as a complement inhibitor at higher concentrations (29–58 µM) [39].

Finally, in contrast to many other antimicrobial peptides, such as β-defensins [40, 41] and polymyxin B [32], arenicins are very stable AMPs that retain their antibacterial activity at high salt concentrations [42] and under high temperature conditions. A property probably due to the presence of one or two disulfide bonds along with several hydrogen bonds, allowing the formation of a ring that contributes to the conformational stability of the molecule (Fig. 1) [33, 43]. Considering that salt-induced inactivation of host defense peptides is an important issue in CF patients [44, 45], the rapid and efficient killing of pathogenic bacteria in the presence of NaCl concentrations makes arenicin peptides particularly interesting lead structures for drug development.

From such physicochemical properties, together with their promising antibacterial activities against four pathogens commonly isolated from CF patients, namely *Pseudomonas. aeruginosa*, *Staphylococcus aureus*, *Bacillus subtilis*, and the yeast *C. albicans* [33]; we hypothesized that these three arenicin isoforms might be active against *M. abscessus*, the most drug-resistant mycobacterial species.

Hence, in this study we first compared the antimycobacterial activity, cytotoxicity and hemolytic activity of **Ar-1**, **Ar-2** and **Ar-3**, as well as their respective disulfide-free derivatives (Fig. 1), with the aim of understanding the role of disulfide bridges in peptide function. To achieve this goal, the selective substitution of Cysteine residues by alpha-amino-*n*-butyric acid (Abu) was performed. Although the effects of replacing half-cystine residues in peptides and proteins with non-bridging residues such as alanine, valine, serine or threonine have been extensively studied, Abu has proved to be a more attractive substitutable residue because it is isosteric to half-cystine and has a similar polarity [46]. As a result, such a Cys to Abu replacement has become the classic strategy used for decades to prevent the formation of disulfide linkage while leaving the peptide sequence globally unaltered [47–49]. We further evaluated the potential of **Ar-1** to permeabilize bacterial cytoplasmic membranes upon interaction with *M. abscessus* cells, and also investigated its binding affinity with monolayers of total lipids from *M. abscessus*.

## Methods

### Mycobacterium* abscessus* strains, growth conditions and arenicin peptides

*Mycobacterium abscessus* S and R strains (CIP 104536^ T^) were grown in Middlebrook 7H9 medium supplemented with 0.05% (*v/v*) Tween 80, 0.2% (*v/v*) glycerol and 10% (*v/v*) oleic acid–albumin–dextrose–catalase (OADC enrichment; BD Difco) (7H9-S^OADC^) at 37 °C under stirring (200 rpm). We used **Ar-1** (RWC^3^VYAYVRVRGVLVRYRRC^20^W) and **Ar-1-Abu**, in which the cysteines in position 3 and 20 were replaced by alpha-amino-*n*-butyric acid (Abu) residues. We also tested **Ar-2** (RWC^3^VYAYVRIRGVLVRYRRC^20^W), **Ar-2-Abu** (cysteines 3 and 20 replaced), **Ar-3** (GFC^3^WYVC^7^VYRNGVRVC^16^YRRC^20^N), **Ar-3-Abu** (all four cysteines were replaced by Abu), **Ar-3(3–20)** (cysteines 7 and 16 replaced), and **Ar-3(7–16)** (cysteines 3 and 20 replaced). All arenicins and arenicin-Abu derivatives were obtained from GenScript (Piscataway, NJ, USA) with purity higher than 95%.

### Minimal inhibitory concentration (MIC) determination via the resazurin microtiter assay

For determining the antimicrobial activity of the different peptides, the Middlebrook 7H9 broth microdilution method was used in sterile 96-well flat-bottom Greiner Bio-One polypropylene microplates with lid (Thermo Fisher Scientific) using the resazurin microtiter assay (REMA) [50–52]. Briefly, *Mycobacterium abscessus* was cultured up to mid log-phase (OD_600_ ~ 1.5–2) and diluted to a cell density of 5 × 10^6^ cells/mL in 7H9-S^OADC^. Then, 100 µL of this above inoculum was distributed to each well containing serial two-fold dilutions of arenicin, arenicin derivative or controls to a final volume of 200 µL. Growth controls containing no peptide (i.e., bacteria only = *B*), inhibition controls containing 50 µg/mL kanamycin (Euromedex, Souffelweyersheim, France) and sterility controls (i.e., medium only) without bacteria were included. After 5 days incubation at 37° C in a humidity chamber to prevent evaporation, 20 µL of a 0.025% (*w/v*) resazurin solution was added to each well. The microplate was re-incubated until appearance of a color change from blue to pink in the control well (i.e., bacteria without antibiotics). Fluorescence of the metabolite resorufin (λ_ex_/λ_em_ = 530/590 nm) was measured using a Tecan Spark 10 M ™ multimode microplate reader (Tecan Group Ltd, France). Relative fluorescence units (RFU) were defined as: RFU% = (test well FU/mean FU of control *B* wells) × 100. MIC_50_ and MIC_90_ values were determined by fitting the RFU% sigmoidal dose–response curves in Kaleidagraph 4.2 software (Synergy Software, Reading, PA). The concentration of peptide leading to 50% and 90% inhibition of *M. abscessus* growth was defined as the MIC_50_ and MIC_90_, respectively. All experiments were performed independently at least three times.

### Cytotoxic assay on human cells

The toxicity of each peptide was evaluated on human cell lines using a resazurin assay as previously described [53, 54]. Human lung epithelial cells BEAS-2B (ATCC CRL-9609), liver cells HepG2 (ATCC HB-8065), and kidney cells A498 (ATCC HTB-44) were used. All cells were cultured in Dulbecco's modified essential medium (DMEM) supplemented with 10% heat-inactivated fetal calf serum (FBS, Thermo Fisher Scientific), and 1% L-glutamine (Thermo Fisher Scientific) (DMEM^FBS^) at 37 °C in a 5% CO_2_ incubator to subconfluent concentrations. Cells were detached with trypsin–EDTA solution (Thermo Fisher Scientific), and around 1 × 10^4^ cells/well were seeded in 96-well flat-bottom microplates (Greiner Bio-One) with a lid, and cultured for additional 48–72 h at 37 °C and 5% CO_2_ until cell confluence. The medium was removed, and 100 µL of serial two-fold dilutions of the peptides in DMEM^FBS^ were added to each well. Following a 48 h-incubation period at 37 °C and 5% CO_2_, cell viability was evaluated using the resazurin assay as described above. Fluorescence of the resazurin metabolite resorufin (λ_ex_/λ_em_ = 530/590 nm) was measured after 2 h incubation at 37 °C and 5% CO_2_ in the dark, with a microplate reader (Biotek, Synergy Mx, Colmar, France). The cytotoxic concentration of peptide leading to 50% cell death as compared to the control was defined as the CC_50_, and was determined by fitting the RFU% sigmoidal dose–response curves in Kaleidagraph 4.2 software (Synergy Software). All experiments were conducted in triplicate.

### Hemolytic activity assay

The hemolytic activity of each peptide was determined as previously described [55]. Briefly, human erythrocytes (red blood cells, hRBC; Divbioscience, Ulvenhout, Netherlands) were washed 2-times with sterile phosphate buffered saline (PBS; Thermo Fisher Scientific) and pelleted by centrifugation at 800 rpm for 5 min; then resuspended in DMEM^FBS^ to a final erythrocyte concentration of 8% (*v/v*). Then, 100 µL of this hRBC solution was distributed to each well of sterile 96-well flat-bottom microplates (Greiner Bio-One) containing serial two-fold dilutions of arenicin, arenicin-Abu derivative or controls in DMEM^FBS^ to a final volume of 200 µL. After 1 h incubation at 37 °C, microplates were centrifuged at 800 rpm for 5 min. Then, 100 µL of supernatant were carefully transferred to a new 96-well microplate, and OD_405_ corresponding to the release of hemoglobin was measured using a microplate reader (Biotek, Synergy Mx, Colmar, France). DMEM^FBS^ and Triton-X100 at 0.1% (*v/v*) were used as negative (*i.e.*, 0% hemolysis) and positive (i.e., 100% hemolysis) controls, respectively. The concentration of peptide leading to 10% and 50% of hemolysis compared to the positive control was defined as the HC_10_ and HC_50_, respectively, and was determined by fitting the dose–response curves in Kaleidagraph 4.2 software (Synergy Software). All experiments were done in triplicate.

### Membrane permeabilization assay

For evaluating whether **Ar-1** induces *M. abscessus* membrane permeabilization, bacterial cells in exponential growth phase were resuspended at 10^9^ cells/mL in 7H9-S^OADC^, before addition of 1 mg/mL propidium iodide (Sigma Aldrich, Lyon, France), a DNA/RNA probe that is cell-impermeable. Then, 100 µL of this mix were distributed in the wells of a 96-well flat-bottom Greiner Bio-One polypropylene microplate already containing **Ar-1** at 4 × MIC_90_, 150 µM cetyltrimethylammonium bromide (CTAB) as positive control, or 7H9-S^OADC^ for the negative control [56]. Fluorescence (λ_ex_/λ_em_ = 530/590 nm) was recorded at 5, 15, 30, 60 and 120 min with a Tecan Spark 10 M ™ multimode microplate reader (Tecan Group Ltd, France). Results were expressed in percentage of permeabilization compared to the maximum fluorescence obtained with CTAB, after removing the fluorescence of the negative control. All experiments were done independently at least three times.

### Lipid insertion assay through surface pressure measurement

The insertion of **Ar-1** into total lipids from *M. abscessus* was measured using reconstituted lipid monolayer at the air–water interface as previously described [56]. Briefly, total lipids from *M. abscessus* S or R dry extracts were incubated at room temperature and under shaking with CHCl_3_-MeOH (1:2; *v/v*) for 16 h. Re-extraction of the residual lipids was done first with CHCl_3_-MeOH (1:1; *v/v*), and then with CHCl_3_-MeOH (2:1; *v/v*) during 16 h. The organic phases were pooled, concentrated under reduced pressure, re-suspended in CHCl_3_-MeOH (3:1; *v/v*), and then washed with 0.3% (*w/v*) NaCl solution. Centrifugation at 5000 rpm for 15 min allowed separation of the organic and aqueous phases. Then, the organic phase was dried over MgSO_4_. Extracted total lipids were dried, solubilized in CHCl_3_-MeOH (2:1, *v/v*), and stored at − 20 °C under nitrogen.

Surface pressure measurement was performed in a controlled atmosphere at 20 ± 1 °C using a fully automated microtensiometer (µTROUGH SX, Kibron Inc., Helsinki, Finland). This apparatus allowed the recording of the adsorption kinetics of a ligand onto a monomolecular film using a set of specially designed Teflon troughs. A few microliters of each total lipid extracts were spread using a 50 µL Hamilton’s syringe at the surface of a sterile ultra-pure water (volume 800 µL) until the desired initial surface pressure (Π_i_) was reached, thus generating a stable lipid monolayer at the air–water interface. The waiting time for the spreading solvent evaporation and for the lipid monolayer to reach equilibrium varied from 5 to 10 min depending on the spreading volume and the initial surface pressure. **Ar-1** was further injected into the ultra-pure water sub-phase below the stable monolayer, with a 10 µL Hamilton’s syringe. The surface pressure increase (ΔΠ_max_) due to the insertion of the peptide into the monolayer of total lipids was then continuously recorded until the equilibrium surface pressure was reached. The data were analyzed using the Filmware 2.5 program (Kibron Inc., Helsinki, Finland).

### Electron microscopy

Cultures of *M. abscessus* S (10^9^ cells/mL) were incubated with **Ar-1** at 4 × MIC_90_ for 120 min, then fixed with a solution of 2% glutaraldehyde in 200 mM HEPES (Thermo Fisher Scientific) (pH 7) directly in the culture medium (*v/v*) for 20 min at room temperature. After centrifugation and removal of the supernatant, the pellet was immediately covered with a second solution of 1% glutaraldehyde for 1 h at 4 °C. After four rinses of 5 min each with the HEPES buffer, the samples were post-fixed with 1% OsO_4_ (Electron Microscopy Sciences, Hatfield, PA, USA) in H_2_O for 1 h at 4 °C. Then, four rinses of 5 min each were done with water. At this stage, the samples were gradually dehydrated using acetone–water mixtures of 50:50, 70:30, 90:10, and 96:4 (*v/v*), respecting a 5 min pause between each bath. Subsequently, the samples were transferred to 100% acetone and placed on a circular coverslip to complete the dehydration process. The coverslip was glued onto a nail-shaped support. Following carbonization, the samples were observed using the scanning electron microscope Tescan VEGA3 SBH (30 kV; Tescan, Brno—Kohoutovice, Czech Republic) and Jeol JSM-7900F (5 kV, 6 mm, LED detector) for higher magnification (Jeol, Croissy-sur-Seine, France).

### Induction of resistance assay

Induction of bacterial resistance by **Ar-1** was achieved by exposure of *M. abscessus* S and R to **Ar-1** or to the control antibiotic clarithromycin (CLR) for 42 consecutive days, and with repeated broth microdilution susceptibility testing and subsequent MIC determination. After each MIC determination, the well containing the highest concentration of **Ar-1** or CLR that allows bacterial growth was diluted 1:1000 in 7H9-S^OADC^ and used as inoculum for the next MIC assay with fresh amounts of **Ar-1** or CLR.

### Statistical analysis

Graphpad Prism 8 (GraphPad Inc.) was used to perform all statistical analyses, which details are given in each figure legend. Differences were considered significant when the calculated *p*-values were smaller than 0.05.

## Results

### Antimicrobial activity and cytotoxicity

**Fig. 2:**
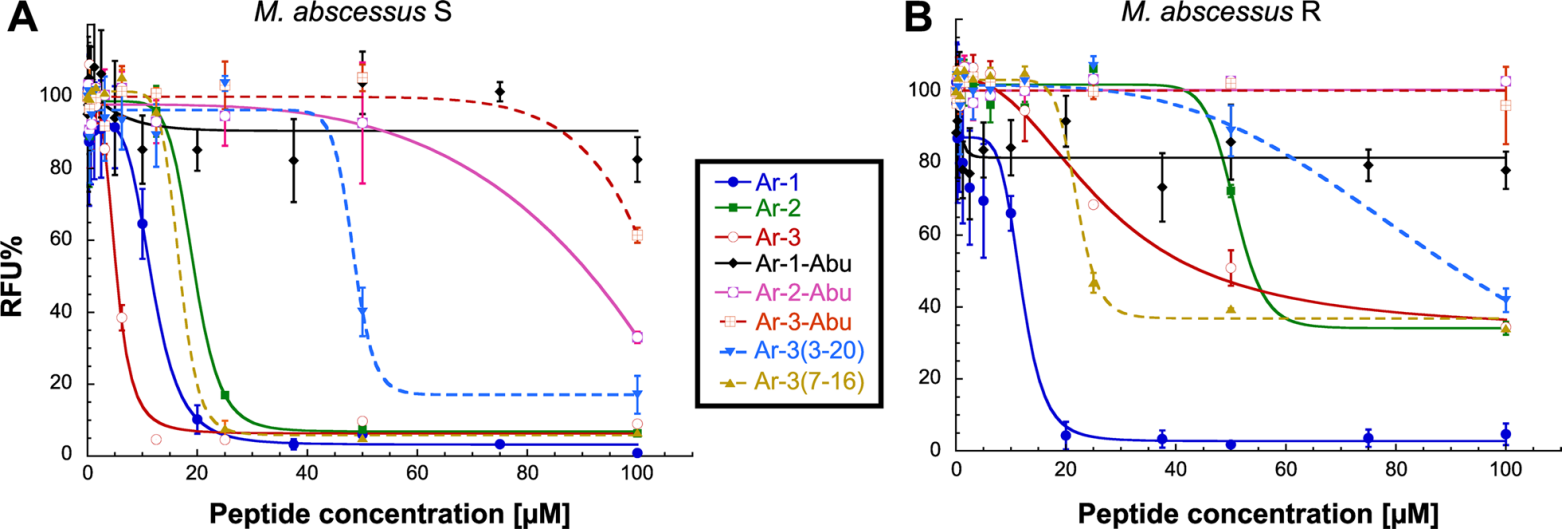
Dose–response activity of the different arenicin and arenicin-Abu peptides against **A** *M. abscessus* S and **B** *M. abscessus* R strains replicating in broth medium, expressed as normalized relative fluorescence units (RFU%). Results are expressed as mean ± SD of at least three independent assays (n ≥ 3)

Drug susceptibility testing of the arenicin and arenicin-Abu derivatives was assessed against both S and R variants of *M. abscessus*, with amikacin (AMK) as standard drug. The corresponding MIC_50_/MIC_90_ values for each peptide, as determined by the REMA assay [51], are reported in Table 1. The three arenicin peptides displayed comparable antibacterial activity against *M. abscessus* S growth, with MIC_50_/MIC_90_ values in the range 5.3–19.8 µM and 12.2–29.3 µM, respectively (Table 1 and Fig. 2). Considering the MIC values reached on *M. abscessus* R, they are 2.5- to eightfold greater for **Ar-2** and **Ar-3**, respectively, than those obtained on the S variant (Table 1). In contrast, only **Ar-1** was found to exhibit similar highly potent MIC_50/90_ values against the two morphotypes (mean MIC_50_ = 11.5 ± 1.0 µM/mean MIC_90_ = 18.8 ± 0.9 µM). Furthermore, among the three native arenicin peptides tested, the fact that **Ar-1** also displayed MIC_90_ values comparable to those of the reference drug amikacin (AMK), made it the best growth peptide inhibitor of the two variants S and R of *M. abscessus*.

**Table 1:** Antibacterial activities (µM) of arenicin and arenicin-Abu derivatives against *M. abscessus* S and R compared to standard drug Amikacin^a^

	MIC_50_/MIC_90_ (µM)	
	*M. abscessus* CIP 104536^ T^	
	S variant	R variant
**Ar-1**	11.6 ± 1.0/20.2 ± 1.6	11.4 ± 0.5/17.5 ± 0.8
**Ar-1-Abu**	> 100	> 100
**Ar-2**	19.8 ± 1.3/29.3 ± 0.6	53.1 ± 0.3/ > 100
**Ar-2-Abu**	89.3 ± 9.0/ > 100	> 100
**Ar-3**	5.3 ± 0.3/12.2 ± 0.1	44.7 ± 3.0/ > 100
**Ar-3-Abu**	> 100	> 100
**Ar-3(3–20)**	48.3 ± 1.4/ > 100	77.8 ± 10.1/ > 100
**Ar-3(7–16)**	17.2 ± 2.7/21.3 ± 3.6	24.6 ± 0.5/ > 100
**AMK**	3.9 ± 0.19 / 5.8 ± 0.20	7.4 ± 0.26/10.1 ± 0.45

All values are expressed as mean ± SD (n ≥ 3). AMK: amikacin

^a^MIC_50_/MIC_90_: peptide minimal concentration leading to 50% and 90% inhibition of in vitro growth, respectively, as determined by the REMA assay

Replacement of all cysteines by Abu residues in the **Ar-1-Abu**, **Ar-2-Abu** and **Ar-3-Abu** resulted in a total loss of their antibacterial activity against the two *M. abscessus* variants (Table 1 and Fig. 2). Interestingly, the selective replacement of only two cysteines by Abu residues in **Ar-3**, likely to remove only one disulfide bond out of two, led to two different antibacterial behaviors. While **Ar-3(7–16)** exhibited MIC_50/90_ values in the same order of magnitude as **Ar-3** against both *M. abscessus* morphotypes, a significant increase of around 10 times was observed for **Ar-3(3–20)** MIC values (MIC_50_ = 48.3 µM/MIC_90_ > 100 µM *vs*. MIC_50_ = 5.3 µM/MIC_90_ = 12.2 µM, for **Ar-3(3–20)** and **Ar-3**, respectively) against *M. abscessus* S, with no real change in its antibacterial activity against the R variant compared to **Ar-3** (Table 1 and Fig. 2). These results highlight the importance of the disulfide bonds (one for **Ar-1**/**Ar-2** and two for **Ar-3**) which in such small peptides enables the integrity of the 3D structure to be maintained, and suggest that their presence is crucial for the antimycobacterial activity of each peptide against *M. abscessus*.

**Fig. 3:**
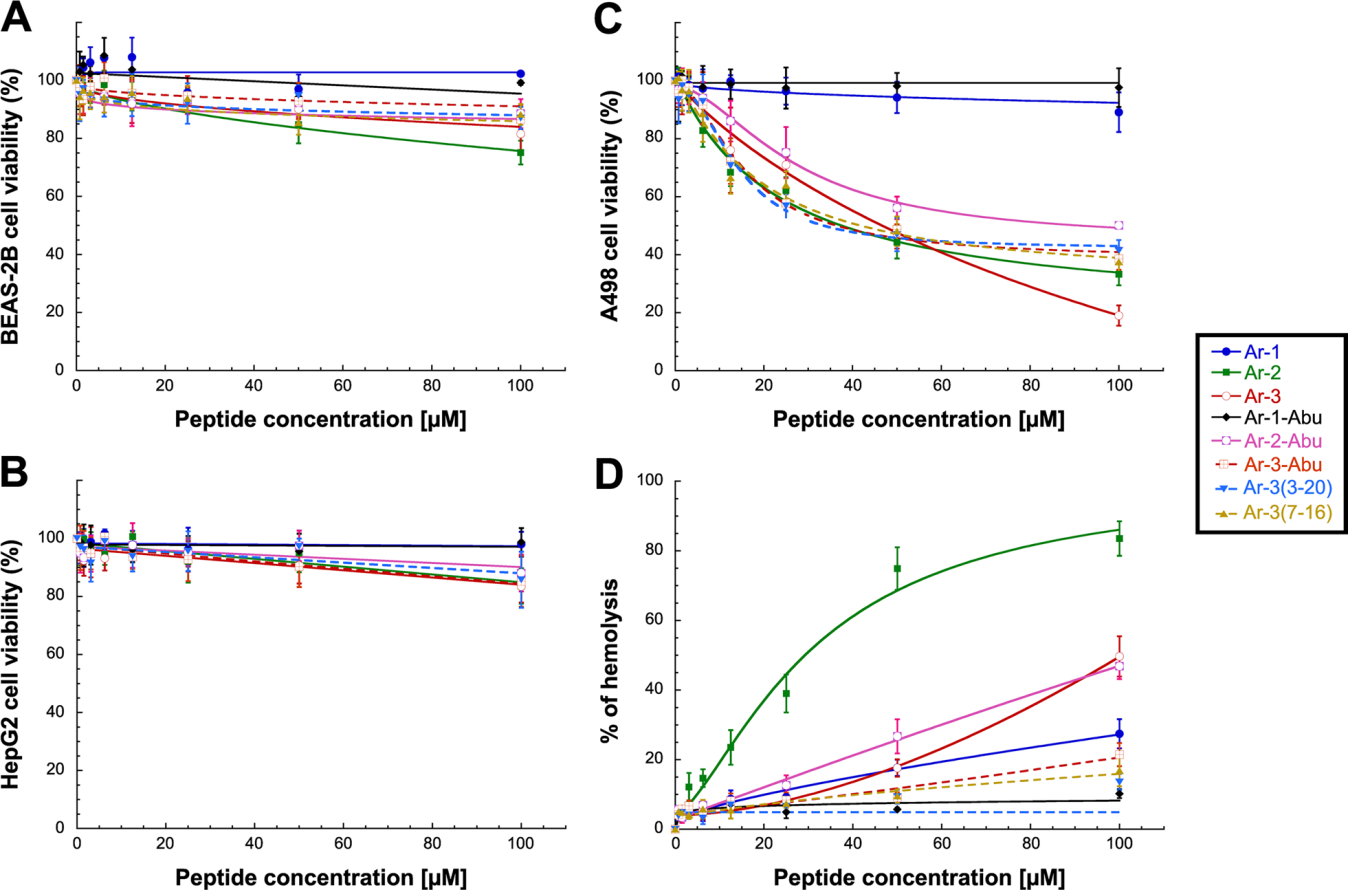
Cytotoxic and hemolytic activity of the arenicin and arenicin-Abu peptides. **A**–**C** Cytotoxicity dose–response curves following 48 h-exposure to increasing concentrations of each peptide on human **A** BEAS-2B cells, **B** HepG2 cells, and **C** A498 cells. **D** Dose–response curves for human erythrocyte hemolysis after exposure to increasing concentrations of each peptide. Results are expressed as the mean ± SD of three independent experiments

In parallel, the cytotoxic activity of the different peptides towards nontumorigenic human lung epithelial cells (BEAS-2B cells), human cells from a hepatocellular carcinoma (HepG2), and human kidney cells from a kidney cancer patient (A498) was investigated using a classical dose–response assay [57]. The calculated response parameter was CC_50_, which corresponds to the concentration required to elicit a 50% decrease in cell viability in vitro, compared to the control. As seen in Table 2 and Fig. 3A, B, no toxicity towards BEAS-2B and HepG2 cells was observed, as demonstrated by CC_50_ values > 100 µM. Regarding kidney A498 cells, although **Ar-1** and **Ar-1-Abu**, and to a lesser extend **Ar-2-Abu** (CC_50_ = 92.1 µM), were nontoxic, all other peptides displayed high toxicity with CC_50_ values ranging from 36.6 to 46.7 µM (Table 2 and Fig. 3C). Such toxicity towards A498 cells was best characterized by calculating the respective therapeutic index [58] (TI_A498_); *i.e.*, the ratio between CC_50_ on A498 cells and MIC_50_ on *M. abscessus* strains, for each peptide (Table 2). Accordingly, and except for **Ar-1** (TI_A498_ > 8.6 for both variants) and **Ar-3** (TIA_498_ = 8.8 on *M. abscessus* S), all tested arenicin and arenicin-Abu peptides displayed TI_A498_ values < 2.2 synonymous with a very narrow window between efficacy and toxicity.

**Table 2:** Evaluation of the cytotoxicity of the arenicin and arenicin-Abu peptides

	CC_50_ (µM)^*a*^			TI_A498_^*b*^	
	BEAS-2B	HepG2	A498	S variant	R variant
**Ar-1**	> 100	> 100	> 100	> 8.6	> 8.8
**Ar-1-Abu**	> 100	> 100	> 100	–	–
**Ar-2**	> 100	> 100	37.7 ± 1.1	1.9	0.28
**Ar-2-Abu**	> 100	> 100	92.1 ± 2.3	1.0	< 1.0
**Ar-3**	> 100	> 100	46.7 ± 1.7	8.8	1.0
**Ar-3-Abu**	> 100	> 100	36.6 ± 0.9	< 0.4	< 0.4
**Ar-3(3–20)**	> 100	> 100	42.0 ± 1.3	0.87	0.54
**Ar-3(7–16)**	> 100	> 100	37.7 ± 1.2	2.2	1.5

All values are expressed as mean ± SD (n = 3)

^a^CC_50_: Cytotoxic concentration of peptide leading to 50% cell toxicity in vitro, compared to the control

^b^Therapeutic Index (TI) calculated by dividing the CC_50_ value on A498 cell lines by the MIC_50_ reached for each *M. abscessus* morphotype

To better characterize the innocuity of the eight peptides, their hemolytic activity on human erythrocytes was further assessed through determination of their respective HC_50_ and HC_10_ values and calculation of their corresponding therapeutic indexes, TI_H50_ and TI_H10_ (Table 3 and Fig. 2D). First, in our experimental conditions, only **Ar-2** was found to exert a strong hemolytic activity against red blood cells with a HC_50_ of 29.2 µM resulting in TI_H50_ of 1.5 and 0.5 with regards to *M. abscessus* S and R variant, respectively (Table 3 and Fig. 2D). Considering their HC_10_ values, except **Ar-1-Abu** and **Ar-3(3, 20)** totally inactive, all other arenicin and arenicin-Abu peptides tested showed variable hemolytic activities on human erythrocytes, resulting in predominantly low (< 0.4) and up to moderate (1–2.5) therapeutic indexes; with **Ar-1** displaying the most promising TI_H10_ values (Table 3).

**Table 3:** Evaluation of the hemolytic activity of the arenicin and arenicin-Abu peptides against Human erythrocytes

	HC_50_ (µM)^a^	TI_H50_^b^		HC_10_ (µM)^a^	TI_H10_^c^	
		S variant	R variant		S variant	R variant
**Ar-1**	> 100	> 8.6	> 8.8	20.2 ± 1.8	1.0	1.2
**Ar-1-Abu**	> 100	–	–	> 100	-	–
**Ar-2**	29.2 ± 2.4	1.5	0.5	4.9 ± 0.7	0.2	–
**Ar-2-Abu**	> 100	> 1.1	–	15.4 ± 1.0	< 0.2	< 0.2
**Ar-3**	> 100	> 18.9	> 2.2	30.3 ± 2.5	> 2.5	< 0.3
**Ar-3-Abu**	> 100	–	–	39.3 ± 3.2	< 0.4	< 0.4
**Ar-3(3–20)**	> 100	> 2.1	> 1.3	> 100	–	–
**Ar-3(7–16)**	> 100	> 5.8	> 4.1	41.1 ± 4.3	1.9	< 0.4

All values are expressed as mean ± SD (n = 3)

^a^HC10 and HC_50_: hemolytic concentration of peptide leading to 10% and 50% hemolysis in vitro, compared to the control

^b^TI_50**:**_ Therapeutic Index calculated by dividing the HC_50_ value by the MIC_50_ reached for each *M. abscessus* morphotype

^c^TI_10_: Therapeutic Index calculated by dividing the HC_10_ value by the MIC_90_ reached for each *M. abscessus* morphotype

Overall, all these results clearly emphasized the efficacy of **Ar-1** against *M. abscessus* S and R. Indeed, **Ar-1** exhibited the most potent MIC values against both variants of *M. abscessus* (mean MIC_90_ ~ 19 µM—Table 1), was non-toxic to host cells (CC_50_ > 100 µM—Table 2), and showed only very weak hemolytic activity thus exhibiting the best therapeutic indexes related to hemolysis of human erythrocytes (TI_H50_ >  ~ 8.7/TI_H10_ ~ 1.1—Table 3). Given all these findings, amongst the 8 tested peptides, **Ar-1** was therefore found to display the best combination of antibacterial and cytotoxic properties and was then selected for further investigations.

### Mechanism of action

**Fig. 4:**
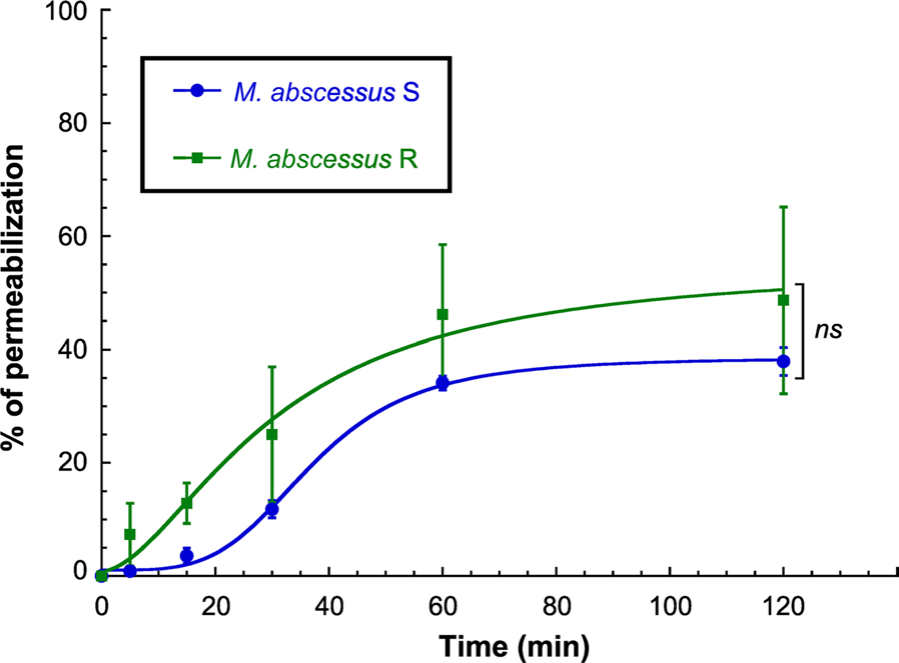
Effect of Ar-1 on *M. abscessus* membrane integrity. *M. abscessus* S (blue circles) or R (green squares) variant was exposed to **Ar-1** at 4 × MIC_90_ concentration. The effect of Ar-1 on the integrity of the mycobacterial membrane was followed over 120 min using propidium iodide and compared to the maximum fluorescence obtained with 150 µM CTAB (100% permeabilization). Results are expressed as mean ± SD of three independent experiments. Statistical analysis was done using a non-parametric Mann–Whitney test using Prism 8.0 (Graphpad Inc.): *ns*, not significant (*p* > 0.05)

The fact that **Ar-1** is known to induce bacterial death through a pore forming mechanism in Gram-negative and -positive bacteria [33, 42, 43, 59–63] prompted us to evaluate its effect on membrane integrity of *M. abscessus* S and R using a propidium iodide assay (Fig. 4). Indeed, propidium iodide is a cell permeable dye which can enter bacteria only if the membrane is damaged, thus forming a highly fluorescent complex with DNA and RNA. As a consequence, the monitoring of propidium iodide fluorescence allows evaluation of membrane integrity. The membranolytic detergent cetyltrimethylammonium bromide (CTAB) was used as a positive control, corresponding to 100% permeabilization [15].

With the two S & R variants of *M. abscessus*, **Ar-1** induced comparable permeabilization pattern over time (Fig. 4). As shown in Fig. 4, a plateau value corresponding to a non-significantly different membrane permeabilization of 36.0 ± 3.0% and 47.4 ± 16.0% (*p* > 0.05) was reached between 60- and 120-min incubation for *M. abscessus* S and R, respectively. Such a result is in line with the similar MIC_50/90_ values obtained by **Ar-1** against the two morphotypes.

### Ar-1 interaction with monolayers of total lipids from *M. abscessus*

**Fig. 5:**
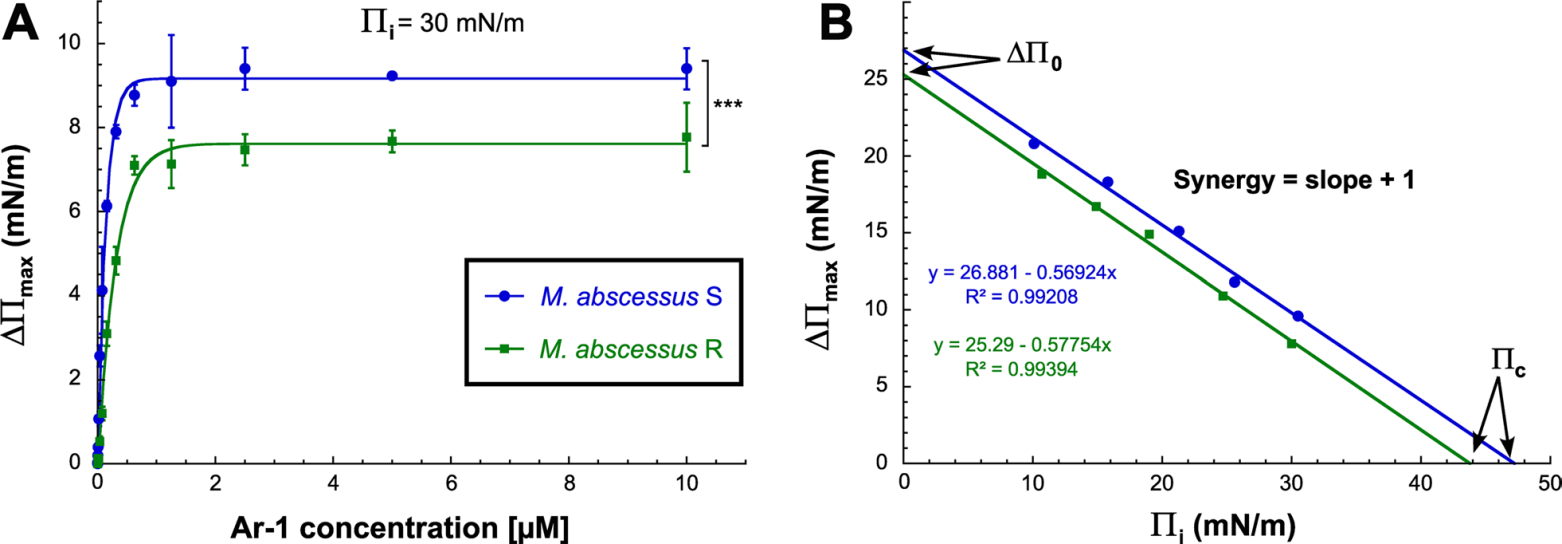
Insertion of Ar-1 into monolayers of total lipids from *M. abscessus*. **A** Variations of the surface pressure increase (ΔΠ_max_) as a function of the peptide concentration upon **Ar-1** insertion into total lipid monolayers from *M. abscessus* S and R. The increase in surface pressure due to the insertion of **Ar-1** into the monolayers of total lipids, spread at an initial surface pressure Π_i_ = 30 mN/m, was continuously monitored. Data are mean values ± SD of three independent experiments. Statistical analysis was done using a non-parametric Mann–Whitney test with Prism 8.0 (Graphpad Inc): ***p < 0.001. **B** Binding parameters of **Ar-1** into a monomolecular film of total lipids from *M. abscessus* S and R. Variation of surface pressure (ΔΠ_max_) after **Ar-1** injection (1.25 µM final concentration) is represented as a function of the initial surface pressure, Π_i_ = 10–30 mN/m

Given the ability of **Ar-1** to cause membrane permeabilization in *M. abscessus*, its ability to insert into monolayers of total lipids from this bacterium [56] was further assessed (Fig. 5). In a first set of experiments, the dose-dependent insertion of **Ar-1** into total lipid monolayers was investigated (Fig. 5A). Total lipids were first spread until an initial surface pressure Π_i_ = 30 ± 0.5 mN/m, corresponding to a lipid packing density theoretically equivalent to that of the outer leaflet of a cell membrane [64]. Increasing concentrations of **Ar-1** were then injected into the aqueous phase below the lipid monolayer, and the variation in surface pressure due to the insertion of the peptide into the total lipid monolayer was continuously recorded until the new equilibrium surface pressure (ΔΠ_max_) was reached (around 90 min—Fig. 5A). In agreement with propidium iodide assay results (Fig. 4), data showed that **Ar-1** was able to insert into total lipid monolayers from both *M. abscessus* S and R with a maximal insertion reached at a concentration of 1.25 µM (*i.e*., ~ 0.11 × MIC_50_), above which a plateau value in terms of ΔΠ_max_ was obtained (Fig. 5A). Moreover, **Ar-1** insertion resulted in a significantly higher equilibrium surface pressure, in the 1.25–10.0 µM concentration range, with total lipid monolayers from *M. abscessus* S (ΔΠ_max_ = 9.3 ± 0.69 mN/m) vs. *M. abscessus* R (ΔΠ_max_ = 7.5 ± 0.65 mN/m;* p*-value < 0.001) (Fig. 5A).

In a second series of experiments, the influence of Π_i_ [65] on the insertion process of **Ar-1** into total lipid monolayers from *M. abscessus* was studied. The optimal final peptide concentration of 1.25 µM, determined above by performing ΔΠ measurements at various **Ar-1** concentrations, was used in these experiments. The plot ΔΠ_max_ = *f*(Π_i_) depicted in Fig. 5B, was first used to evaluate the binding parameters of **Ar-1**. Linear extrapolation to no increase in the surface pressure (ΔΠ_max_ = 0) allowed to determine the critical surface pressure (Π_c_), also named “maximum insertion pressure” [66], as being equal to 47.2 and 43.7 mN/m for *M. abscessus* S and R, respectively. Above this Π_c_ value, which is specifically related to the peptide and the lipid forming the monolayer, no increase in the surface pressure will then occur. In addition, the synergy factor (slope of the linear regression + 1) which has been demonstrated to modulate protein/peptide insertion, as well as ΔΠ_0_ (*y*-intercept of the curve corresponding to Π_i_ = 0) [65–67] can also be deduced from the same plot. Indeed, and as defined by Salesse group [66, 67], the observed positive synergy values (0.42–0.43) and the occurrence of a ΔΠ_0_ value (25.3–26.9 mN/m) lower than the related Π_c_ (43–47 mN/m) are consistent with an ‘insertion’ surface pressure and favorable interactions between the peptide and the monolayer of total lipids from *M. abscessus*.

### Microscopic observations

**Fig. 6:**
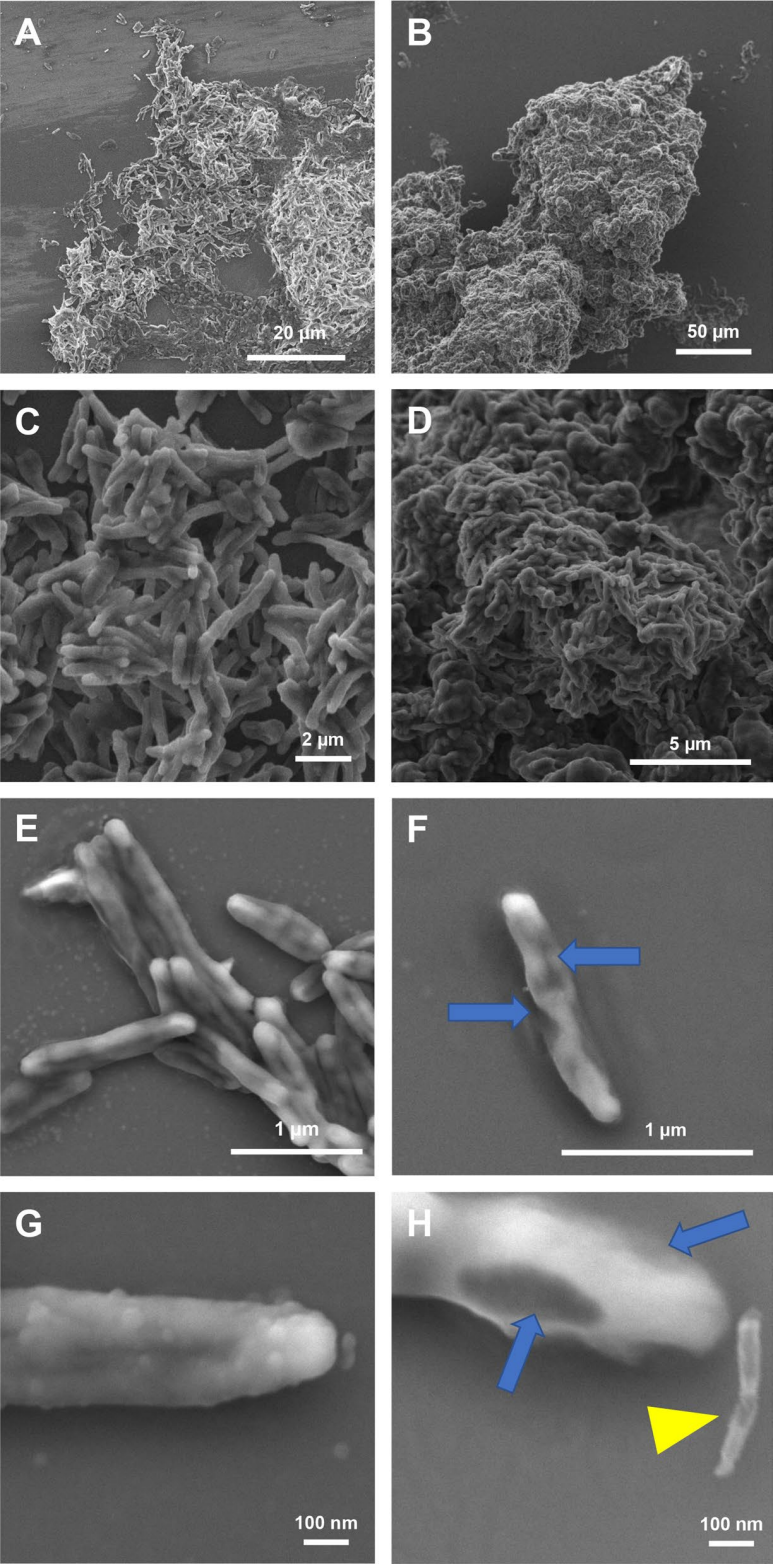
Scanning electron microscopy of untreated control cells (left panels **A**, **C**, **E**, and **G**) and cells treated with **Ar-1** at 4 × MIC_90_ concentration for 120 min (right panels **B**, **D**, **F**, and **H**). Control cells appear well distinguishable, with clear cell contour. Cells treated with **Ar-1**, when in clusters, seem to be covered in a sticky substance (**B** and **D**). When isolated, cells treated with **Ar-1**, contrary to control cells, have blurred edges (blue arrow in **F** and **H**). Some cell pieces can also be seen in the treated sample (yellow arrowhead in **H**), contrary to the control one

*Mycobacterium abscessus* S cultures were treated with **Ar-1** at 4 × MIC_90_ for 120 min, then fixed and processed for scanning electron microscopy experiments. As can be seen in Fig. 6, different modifications in the cell shape could be observed on **Ar-1**-treated *M. abscessus* S cells (Fig. 6B, D, F, and H) as compared to untreated one (Fig. 6A, C, E, and G). Indeed, bacteria incubated with **Ar-1**, especially when they are in clusters, seem to be covered by a sticky substance (Fig. 6B, D vs. A, C). Furthermore, clear alteration of cell wall morphology can be seen on single isolated bacteria treated with **Ar-1** which seem to be empty with edges appearing blurred (see blue arrows in Fig. 6F and H). It should be noted that some cell fragments are also visible (see yellow arrowhead in Fig. 6H), thus strengthening the appearance of empty cells. In contrast, untreated control cells show a smooth and intact bacterial membrane (Fig. 6E and G). All these data clearly support disruption of the bacterial membrane through the pore forming activity of **Ar-1** on *M. abscessus* S cells.

### Induction of resistance assay

**Fig. 7:**
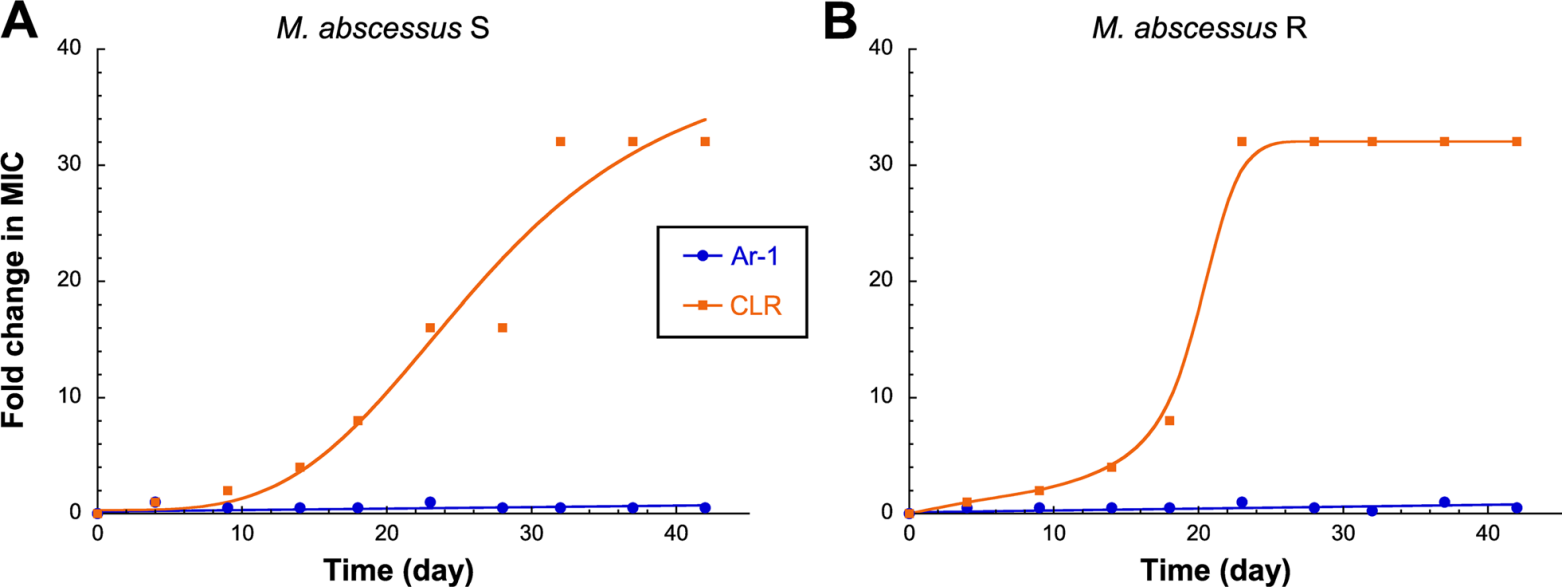
*Mycobacterium abscessus* is not able to develop resistance mechanisms to** Ar-1**. The ability of *M. abscessus* **A** S or **B** R to develop resistance to **Ar-1** was assessed by repeated MIC determination over 42 consecutive days exposure to this peptide. Clarithromycin (CLR) prompted to cause resistance was used as a positive control

Regarding **Ar-1**, all results obtained above suggest that this peptide may target the bacterial membrane of *M. abscessus*. Given such membranolytic activity, **Ar-1** should be less prompt at selecting resistant bacteria compared to conventional antibiotics targeting enzymes/proteins for which mutations may induce resistance. This is especially true with *M. abscessus*, which is naturally resistant to many antibiotics and possesses the ability to acquire reversible and irreversible resistance to various antibiotics after a long period of treatment. In this context, we evaluated whether this mycobacterium could develop resistance to **Ar-1** (Fig. 7).

As depicted in Fig. 7, a 42-days continuous exposure to **Ar-1** induced no significant change in the MIC_90_ value of this peptide against *M. abscessus* S and R variants, contrary to the conventional antibiotic clarithromycin (CLR) for which a 32-fold increase in MIC_90_ values was observed after 20–30 days of exposure.

## Discussion

Infections due to non-tuberculous mycobacteria represent a major public health problem worldwide, including developed countries where their incidence can even be higher than that of tuberculosis [68–70]. Among non-tuberculous mycobacteria, *M. abscessus* is a problem, causing skin infections as well as infections involving soft tissues [71, 72], being increasingly prevalent during the last decade in CF patients [73, 74]. Yet, the treatment usually lasts 18 to 24 months with a cocktail of antibiotics, inducing severe adverse events in patients and also being a high cost for society [70, 75–77]. Furthermore, the treatment outcomes are often disappointing with a cure rate of patients with a *M. abscessus* pulmonary infection of only 25–58% [76, 78, 79]. This can be explained by several characteristics of *M. abscessus*, notably biofilm-like aggregation and intracellular survival, limiting antibiotic efficiency, as well as *M. abscessus* high resistance to many antibiotics [69, 80]. This resistance, whether intrinsic, adaptive or acquired, is such that *M. abscessus* is referred to as the “antibiotic nightmare” [10]. As a consequence, new therapeutic tools, efficient against both *M. abscessus* S and R morphotypes, generating no resistance, are needed.

With no novel antibiotics entering the market in the past twenty years and the emergence of multidrug resistance (MDR) in many pathogenic bacteria, AMPs are making a comeback as the tool of choice against such pathogens. Indeed, thirty years after their discovery, a better understanding of their mode of action, their modification feasibilities and their synthesis at lower cost are reigniting the commercial development of AMPs [20, 81]. Among AMPs, arenicins are β-hairpin peptides stabilized by disulfide bonds which have been evidenced to exert antibiotic activities in vivo in mouse models of urinary tract infection, peritonitis and endotoxemia [82]. Arenicin analogues are currently undergoing preclinical studies against infections caused by Gram-negative and Gram-positive MDR bacteria [35, 83], thus highlighting the relevance of this almost unexplored family of AMPs in comparison with conventional antibiotics.

In this context, we have evaluated the antimycobacterial activity of the three arenicin isoforms; **Ar-1**, **Ar-2** and **Ar-3**; as well as their respective disulfide-free Abu-derivatives; **Ar-1-Abu**, **Ar-2-Abu**, **Ar-3-Abu**, **Ar-3(3–20)** and **Ar-3(7–16)**; on the growth of the S & R variants of *M. abscessus*.

Among these arenicin and arenicin-Abu peptides, we showed that **Ar-1** displayed the best combination of antimycobacterial activity against both morphotypes (MIC_90_ ~ 19 µM), cytotoxicity on eukaryotic cells and hemolytic activity towards human erythrocytes, resulting in relatively good therapeutic indexes [58] (Figs. 2, 3 and Table 1, 2). Concerning **Ar-2** and **Ar-3**, they displayed good antibacterial activity against *M. abscessus* S-variant only, but also showed potent cytotoxicity against A498 cells and hemolytic activity (for **Ar-2**), which considerably limits their use in humans. Interestingly, the replacement of the two bridged Cys^3^ & Cys^20^ residues by Abu led to totally inactive and nontoxic peptides in the case of the **Ar-1-Abu** and **Ar-2-Abu**. Similar results were reported by Lee et al. [60] who found that a linear derivative of **Ar-1,** generated by substituting Cys^3^ & Cys^20^ with serine residues, was fourfold less active against Gram-negative bacterial cells than native **Ar-1**. In contrast, when the two cysteines were replaced by alanine residues, Krenev et al. showed that the resulting Ar-1(C/A) peptide was still able to form a twisted β-hairpin structure despite the absence of its disulfide bond, and also to retain both antibacterial and hemolytic activities as compared to natural peptide [84]. From these biological data, it can be concluded that the hydrophobic-hydrophilic balance, the disulfide bridge, and the amphipathic β-sheet structure of the peptide play important roles in the biological activities of arenicins. Regarding **Ar-3**, neither the selective nor the complete replacement of the four cysteine residues improved the toxicity of the **Ar-3(3–20)**, **Ar-3(7–16)** and **Ar-3-Abu** resulting peptides (Fig. 3 and Table 2). In contrast, the fact that **Ar-3(7–16)** retained similar antibacterial activity to **Ar-3**, whereas **Ar-3-Abu** was inactive and **Ar-3(3–20)** weakly active thus highlighted the importance of Cys^7^-Cys^16^ bridge, which appears to drive the global antibacterial activity of all **Ar-3** peptides probably by maintaining a correct folding of their respective 3D structure.

Such differences, however, between **Ar-1** and **Ar-2**, were surprising as the two isoforms share 95% sequence identity and differ in only one residue: Val^10^ in **Ar-1** and Ile^10^ in **Ar-2** (Fig. 1). Such a single Val to Ile mutation has however been reported in the case of transthyretin transport protein, and has been shown to be responsible for a cardiomyopathy characterized by accelerated cardiac amyloid deposition [85]. In a recent study on the link between arenicins’ structure and antimicrobial and hemolytic activities [84], the authors suggested that the overall 3D conformation of **Ar-1** could be described as more compact compared with the more elongated **Ar-2** conformation. From our data, the specific position of the Val^10^ and Ile^10^ residues at the extremity of one β-sheet of **Ar-1** and **Ar-2**, respectively, proved to be crucial for their inhibitory effect as well as for their cytotoxic and hemolytic activities. This is particularly true for the *M. abscessus* R variant, for which the MIC values of **Ar-2** are ≥ 5 times higher than those of **Ar-1** (Table 1). On the other hand, the effects of **Ar-3** may be explained by its amino acid composition, which differs mainly from **Ar-1** and **Ar-2** with only 47% sequence identity.

Given their very limited application in humans due to lack of significant antibacterial activity combined with high toxicity to A498 cells, the use of Abu derivatives should be limited to comparative mechanistic studies with arenicin peptides. However, it might be interesting to test them on cancer cell lines other than A498 kidney cells to check whether these Abu-peptides have the same effect, which could pave the way for the possibility of a novel treatment for cancer cells.

We further focused on **Ar-1** to better characterize its mechanism of action against *M. abscessus* S and R. Our data showed that this peptide provokes a ~ 42% mean permeabilization of *M. abscessus* membrane regardless of the S or R variant tested (Fig. 4). Moreover, **Ar-1** was also able to efficiently interact and insert into monolayers of total lipids from *M. abscessus* S and R at high surface pressures: it was not excluded from the monolayer, and it exhibited a slightly higher affinity for lipid extracts from the S morphotype (Fig. 5). A recent study has underlined the importance of the surface-exposed glycopeptidolipids (GPL) present only in the S variant in *M. abscessus* hydrophobicity [86]. By using quantitative imaging atomic force microscopy, the authors demonstrated that the transition from a smooth to a rough colony morphology, caused by the loss of GPL, led to a dramatic change in surface hydrophobicity, smooth bacteria displaying unusual nanodomains with varying degrees of hydrophobicity. In the case of **Ar-1**, the presence of GPL in *M. abscessus* S may alter cell-wall fluidity/permeability thus slightly favoring the insertion of this peptide at the origin of membrane permeabilization, cytoplasm leakage and bacterial cell lysis as depicted in Fig. 6.

As previously observed with another class of synthetic copolymers [56], such a pore-forming mode of action is likely to prevent the bacteria from developing any kind of resistance mechanism. This was clearly confirmed by the absence of appearance of **Ar-1** resistance in *M. abscessus* S and R variants after a 42 days continuous exposure to this peptide (Fig. 7).

Overall, the fact that **Ar-1** possesses good bactericidal activity against the S and R morphotypes of *M. abscessus*, without significant toxicity or hemolytic activity and without inducing resistance, is therefore very promising. Furthermore, since CF patients are initially infected with the S variant of *M. abscessus*, which can evolve into the R variant capable of forming cords and leading to severe and almost incurable lung infection, **Ar-1** could represent a valuable alternative strategy to kill *M. abscessus* from the early stage of infection to the chronic lung infections.

## Conclusion

In this article we have confirmed the great interest of **Ar-1**, a 21-residue antimicrobial peptide, in the fight against *M. abscessus* infections. Moreover, the fact that **Ar-1** also exhibits a broad spectrum of activities against Gram-positive and Gram-negative bacterial as well as fungal pathogens [33], such as *Pseudomonas aeruginosa*, *Staphylococcus aureus*, and *Candida albicans*, respectively, either alone or in combination with classical antibiotics as its synergistic activity has also been demonstrated [87], opens new perspectives for CF patients. Future studies should however be conducted, notably in vivo studies, to confirm the interest of this peptide in the therapeutic arsenal against bacterial infections in CF patients. In particular, to circumvent the described low hemolytic activity, encapsulation experiments might be done in order to use this peptide as an aerosol in human therapy, similar to amikacin liposome inhalation suspension [88], rather than injected into patients or administered orally.

## Data Availability

All data generated and analyzed during this study are included in this article.

## Abbreviations

Abu: Alpha-amino-*n*-butyric acid residue
AMK: Amikacin
AMPs: Antimicrobial peptides
Ar: Arenicin
CC_50_: Cytotoxic concentration leading to 50% cell toxicity in vitro
CF: Cystic fibrosis
CLR: Clarithromycin
CTAB: Cetyltrimethylammonium bromide
DMEM: Dulbecco’s modified essential medium
FBS: Fetal calf serum
GPL: Glycopeptidolipids
MIC: Minimal inhibitory concentration
REMA: Resazurin microtiter assay
TI: Therapeutic index
